# Supplementary material for: Microtubules orchestrate local translation to enable cardiac growth
Source: Nat Commun. 2021 Mar 11;12:1547. doi: 10.1038/s41467-021-21685-4 (PMC7952726; doi:10.1038/s41467-021-21685-4)
Supplement: Supplementary file 1 — Supplementary Information [file 41467_2021_21685_MOESM1_ESM.pdf]

Supplementary Information

Microtubules orchestrate local translation to enable cardiac growth

Emily A. Scarborough*, Keita Uchida*, Maria Vogel, Noa Erlitzki, Meghana Iyer, Sai Aung Phyo, Alexey Bogush, Izhak Kehat, Benjamin L. Prosser[#]

* These authors contributed equally to this work, listed alphabetically: Emily A. Scarborough, Keita Uchida

# Address correspondence to Benjamin L. Prosser, bpros@pennmedicine.upenn.edu

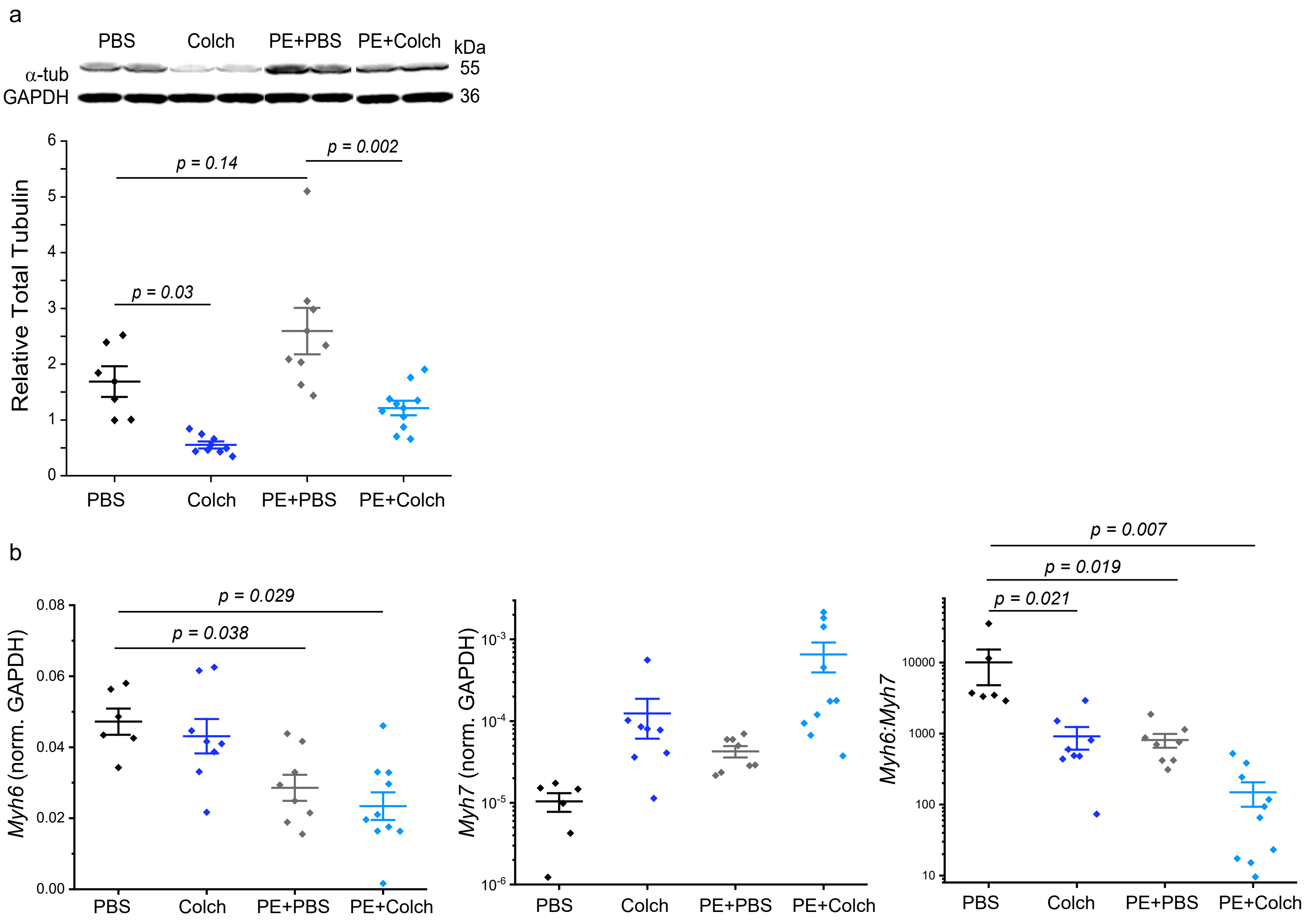

Supplementary Figure 1. Total tubulin levels of PE-induced mouse hypertrophy model

a, (Top) Representative western blot images of α−tubulin and loading control GAPDH; (bottom) Quantification of the total tubulin levels normalized to control samples. b, Quantification of Myh6, Myh7, and Myh6:7 expression ratio by qPCR. For all panels, PBS (N=6), Colch (N=8), PE+PBS (N=8) and PE+Colch (N=10). Statistical significance determined via one-way ANOVA with post hoc Bonferroni comparison. For all graphs shown in figure, the mean line is shown, with whiskers denoting standard error (SE) from the mean. Source Data are provided as a Source Data file.

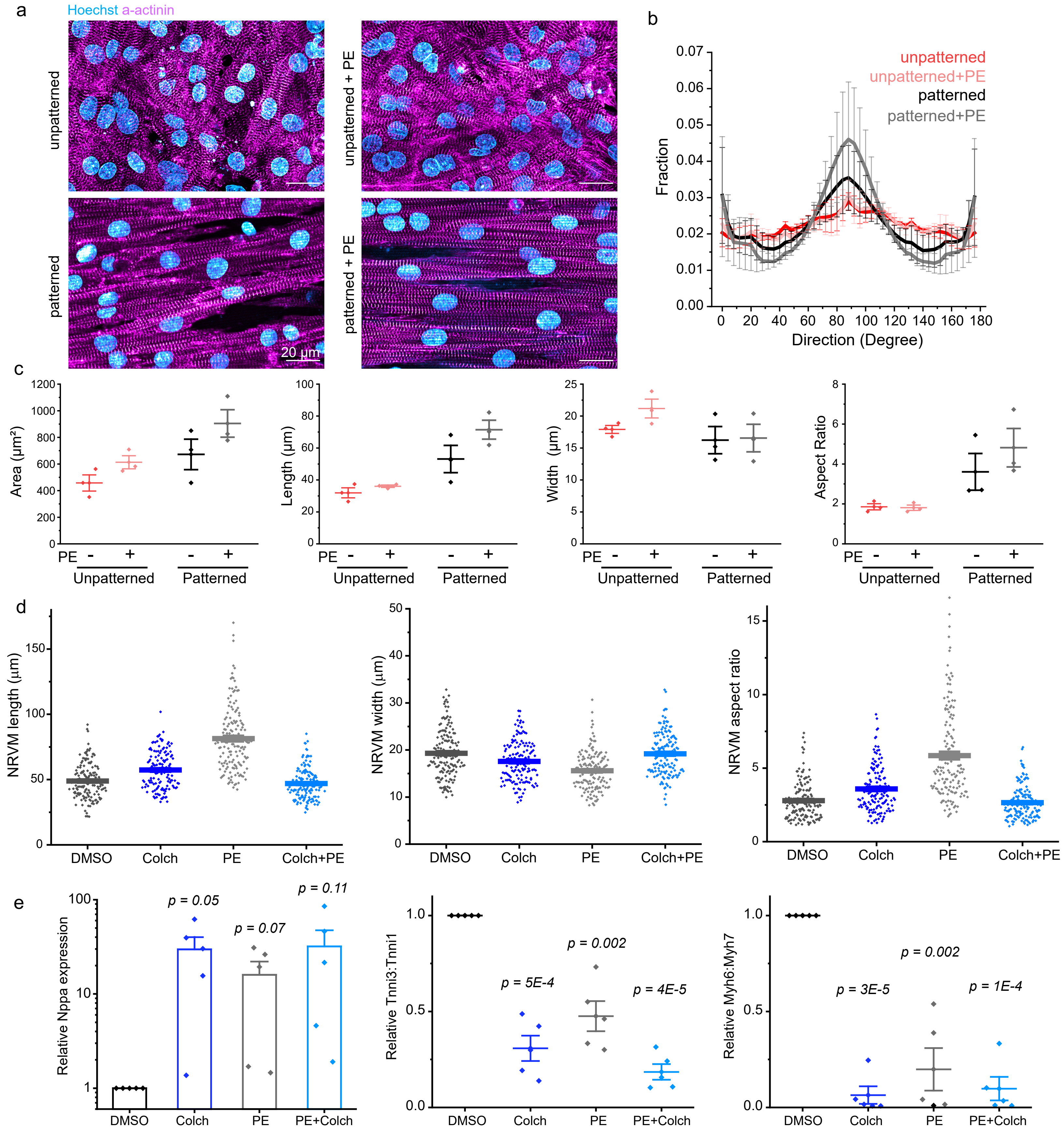

Supplementary Figure 2. Effect of nanopatterning on NRVM morphology and quantification of hypertrophic transcriptional markers

a, Representative immunofluorescence images of NRVMs cultured on unpatterned dishes, unpatterned dishes and treated with PE, patterned dishes, and patterned dishes and treated with PE. **b,** Histogram of α–actinin directionality determined by local gradient orientation. 0 and 180 degrees correspond to the orientation of the patterned substrate. unpatterned (n=90), unpatterned + PE (n=105), patterned (n=88), patterned + PE (n=79). **c,** Cell area, major axis length, minor axis length and aspect ratio measured from live cell images of NRVMs. unpatterned (n=174), unpatterned + PE (n=175), patterned, (n=178), patterned + PE (n=179). **d,** Major axis length, minor axis length, and aspect ratio of live NRVMS on patterned dishes treated with DMSO (n=147), PE (n=148), Colch (n=138) or PE+Colch (n=143). **e,** Relative expression of Nppa (left) and relative ratios of adult to fetal isoforms of sarcomeric proteins (right two graphs) in NRVMs on patterned dishes treated with DMSO, Colch, PE and PE+Colch. N=5. One-sample, two-tailed t-test vs. mean = 1, unadjusted p-value reported. For all graphs shown in figure, the mean line is shown, with whiskers denoting standard error (SE) from the mean. Source Data are provided as a Source Data file.

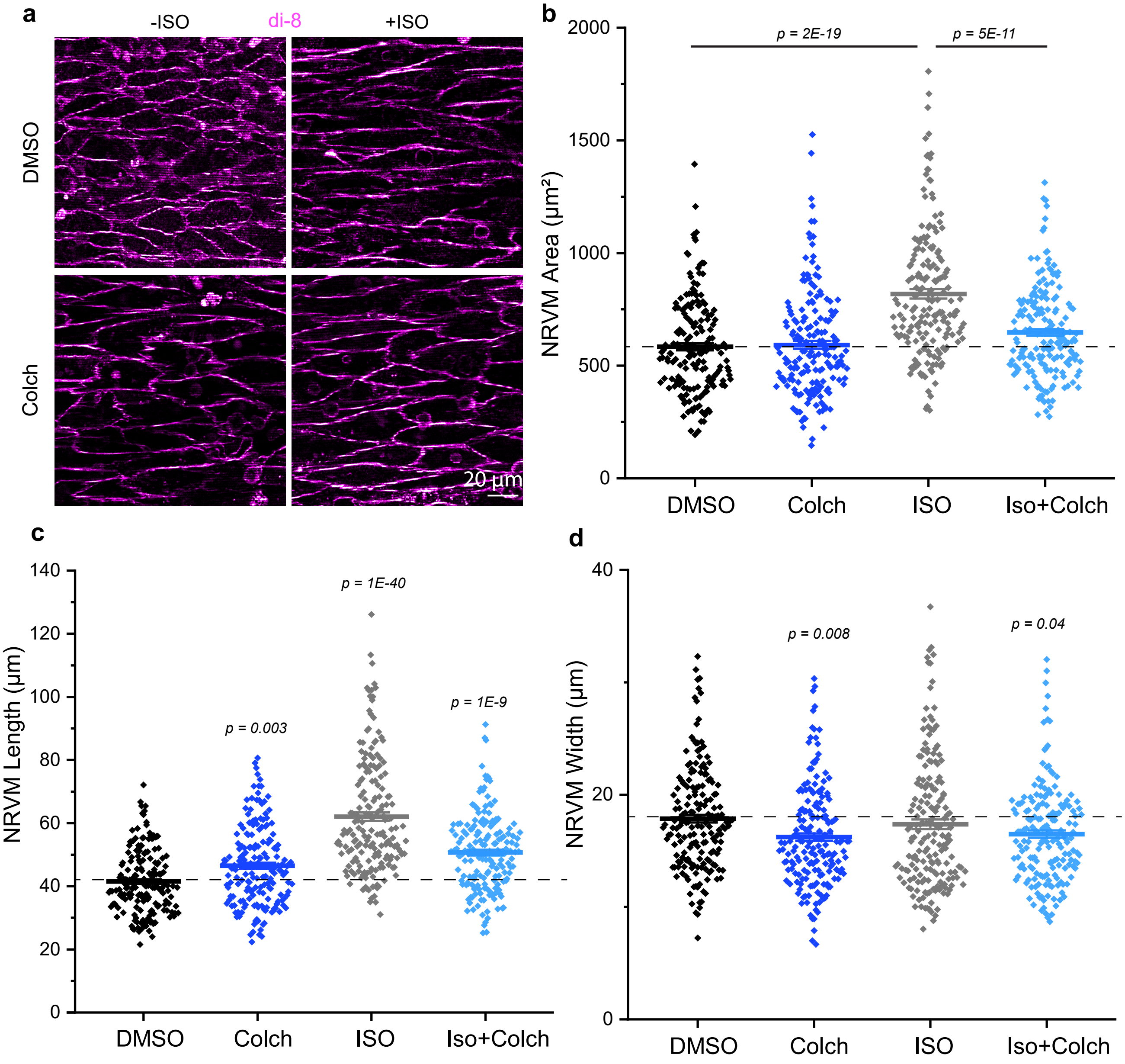

Supplementary Figure 3. Microtubule disruption blocks isoproterenol induced hypertrophy in NRVMs. **a,** Representative live cell images of NRVMs treated with DMSO, ISO, Colch, or ISO+Colch. Quantification of cell area (**b**), cell length (**c**), and cell width (**d**) from DMSO (N=3, n=180), ISO (N=3, n=180), Colch (N=3, n=180), or ISO+Colch (N=3, n=180) treated cells. Statistical significance determined via one-way ANOVA with post hoc Bonferroni comparison. For all graphs shown in figure, the mean line is shown, with whiskers denoting standard error (SE) from the mean. Source Data are provided as a Source Data file.

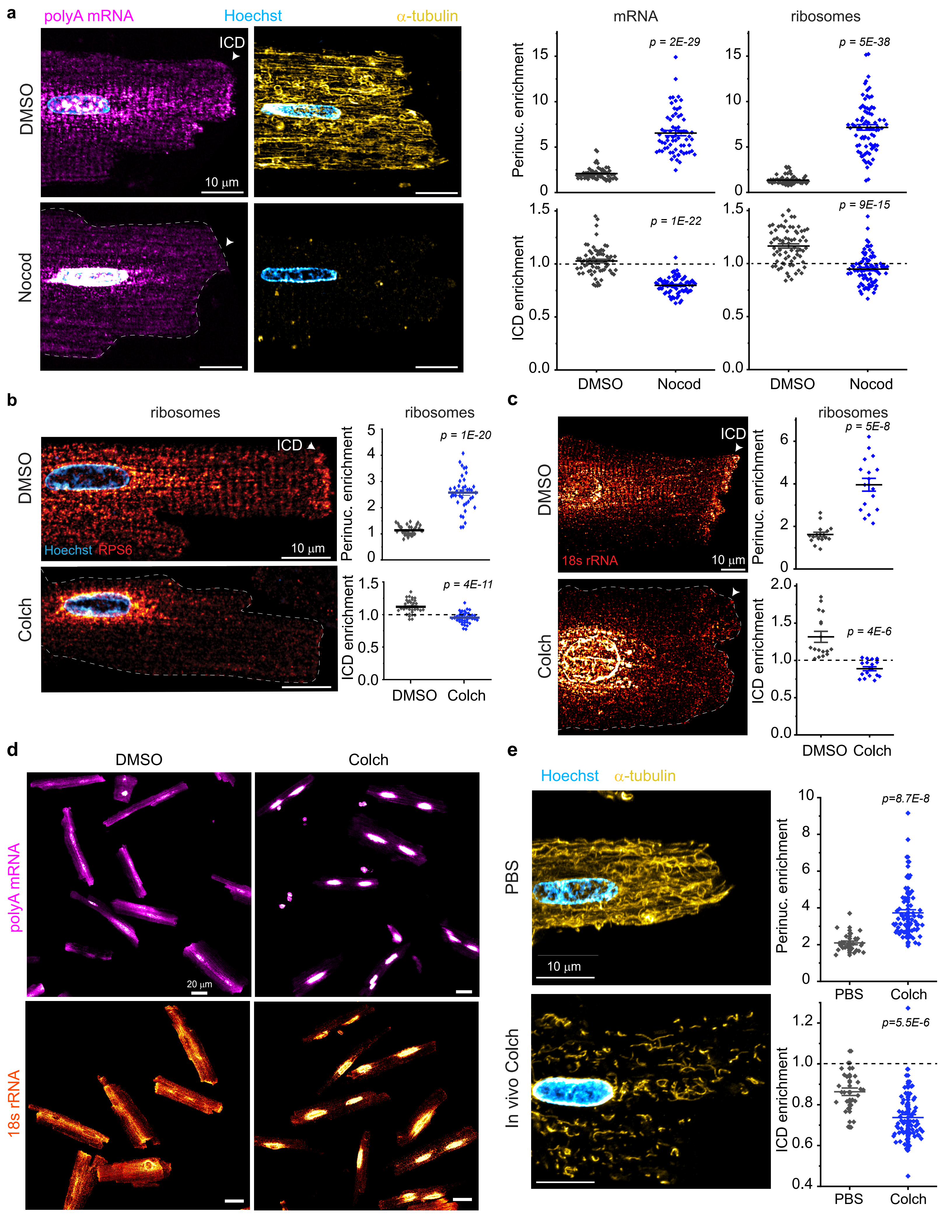

a
polyA mRNA
Hoechst
α-tubulin
ICD
DMSO
Nocod
10 μm
mRNA
ribosomes
Perinuc. enrichment
ICD enrichment
p = 2E-29
p = 5E-38
p = 1E-22
p = 9E-15
DMSO
Nocod
b
ribosomes
Hoechst RPS6
Colch
p = 1E-20
p = 4E-11
c
18s rRNA
p = 5E-8
p = 4E-6
d
20 μm
e
PBS
In vivo Colch
p=8.7E-8
p=5.5E-6

Supplementary Figure 4. Microtubule destabilization mislocalizes mRNA and ribosomes.
a, (left) Representative images of (left column) polyA mRNA and (right column) immunofluorescence in ARVMs treated with (top) DMSO or (bottom) Nocod. White arrow indicates ICD. (right) Nuclear:cytosolic and ICD:cytosolic ratios of polyA mRNA (shown at left) and 18s rRNA mean fluorescence intensities of ARVMs treated with DMSO and Nocod. mRNA: N=2; (DMSO n=65 ;Nocod n=67); ribosomes: N=2; (DMSO n=81 ; Nocod n=72). Statistical significance determined via two sample, two-tailed t-test. **b,** (left) Representative immunofluorescence images of RPS6 in ARVMs treated with DMSO and Colch; (right) Nuclear:cytosolic and ICD:cytosolic ratios of mean RPS6 fluorescence. DMSO (N=2, n=36), Colch (N=2, n=40).
c, (left) Representative images of isolated human cardiomyocytes treated with (top) DMSO or (bottom) Colchicine and hybridized with 18s rRNA. White arrow indicates ICD. (right) Nuclear:cytosolic and ICD:cytosolic ratios of mean 18s rRNA fluorescence. DMSO (N=1, n=16), Colch (N=1, n=17). Statistical significance determined via two sample, two-tailed t-test.
d, Representative smFISH tiled images of DMSO and Colch-treated ARVMs. **e,** (left) Representative immunofluorescence images of tubulin in ARVMs isolated from rats injected with PBS or Colch. (right). Nuclear:cytosolic and ICD:cytosolic ratios of mean polyA fluorescence. PBS (N=1, n=26), Colch (N=3, n=69). Statistical significance determined via one-way ANOVA with post hoc Bonferroni comparison. For all graphs shown in figure, the mean line is shown, with whiskers denoting standard error (SE) from the mean. Source Data are provided as a Source Data file.

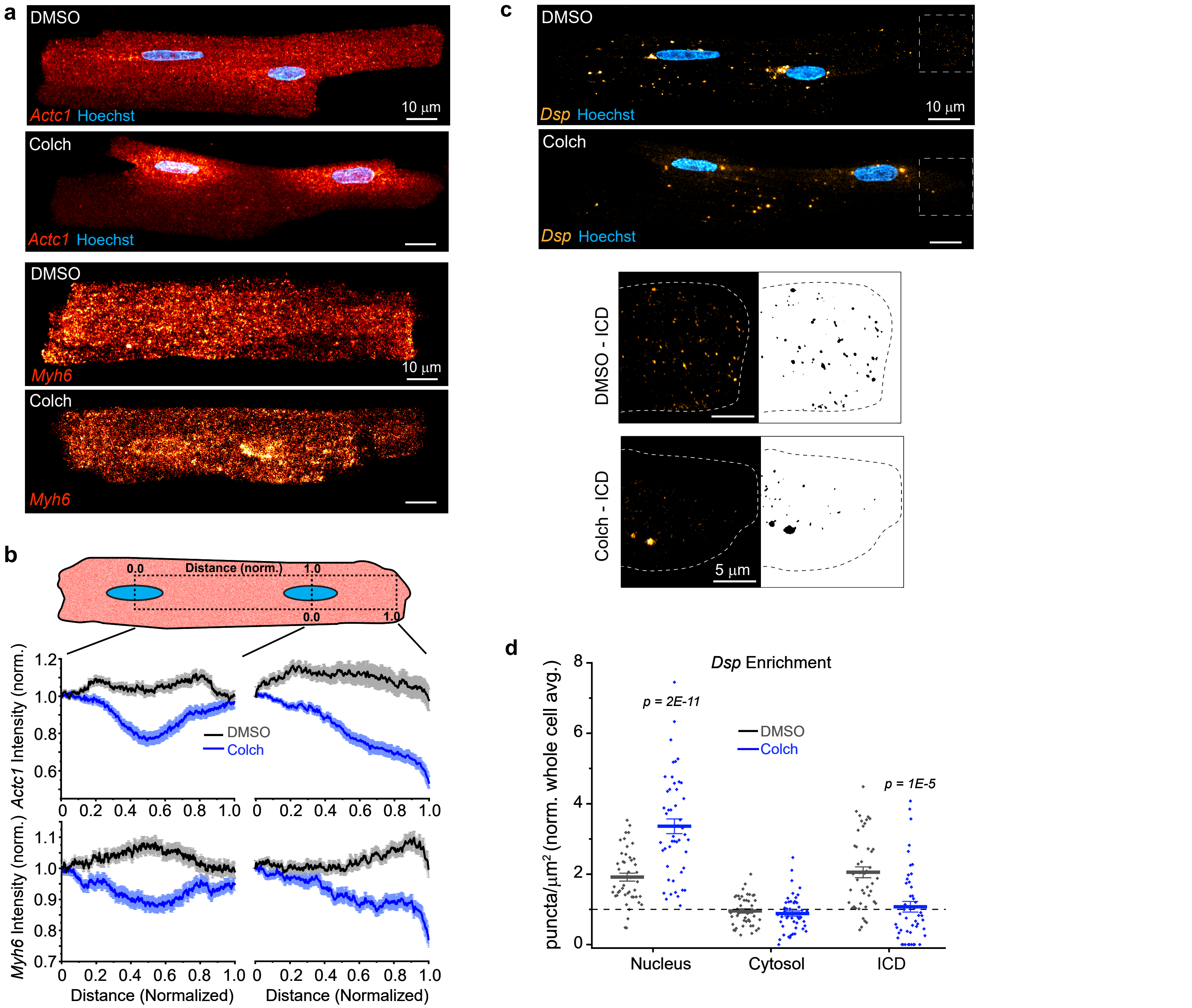

Supplementary Figure 5. Microtubule destabilization mislocalizes specific sarcomeric transcripts. **a,** Representative images of DMSO or Colch-treated ARVMs hybridized for Actc1 and Myh6. **b,** Cartoon (top) depicting placement of line scans drawn for Actc1 and Myh6 image analysis. Normalized Actc1 intensity for DMSO (n=48) or Colch (n=48). Normalized Myh6 intensity for DMSO (n=42) or Colch (n=42). **c,** (top) Representative images of DMSO or Colch-treated ARVMs hybridized for Dsp. (bottom) Zoomed-in regions of Dsp cells with corresponding image thresholded to right. **d,** Dsp puncta/µm2, normalized to whole cell area, for DMSO (n=48) and Colch (n=49). Statistical significance determined via one-way ANOVA with post hoc Bonferroni comparison. All experiments/conditions in figure are three independent biological replicates. For all graphs shown in figure, the mean line is shown, with whiskers denoting standard error (SE) from the mean. Source Data are provided as a Source Data file.

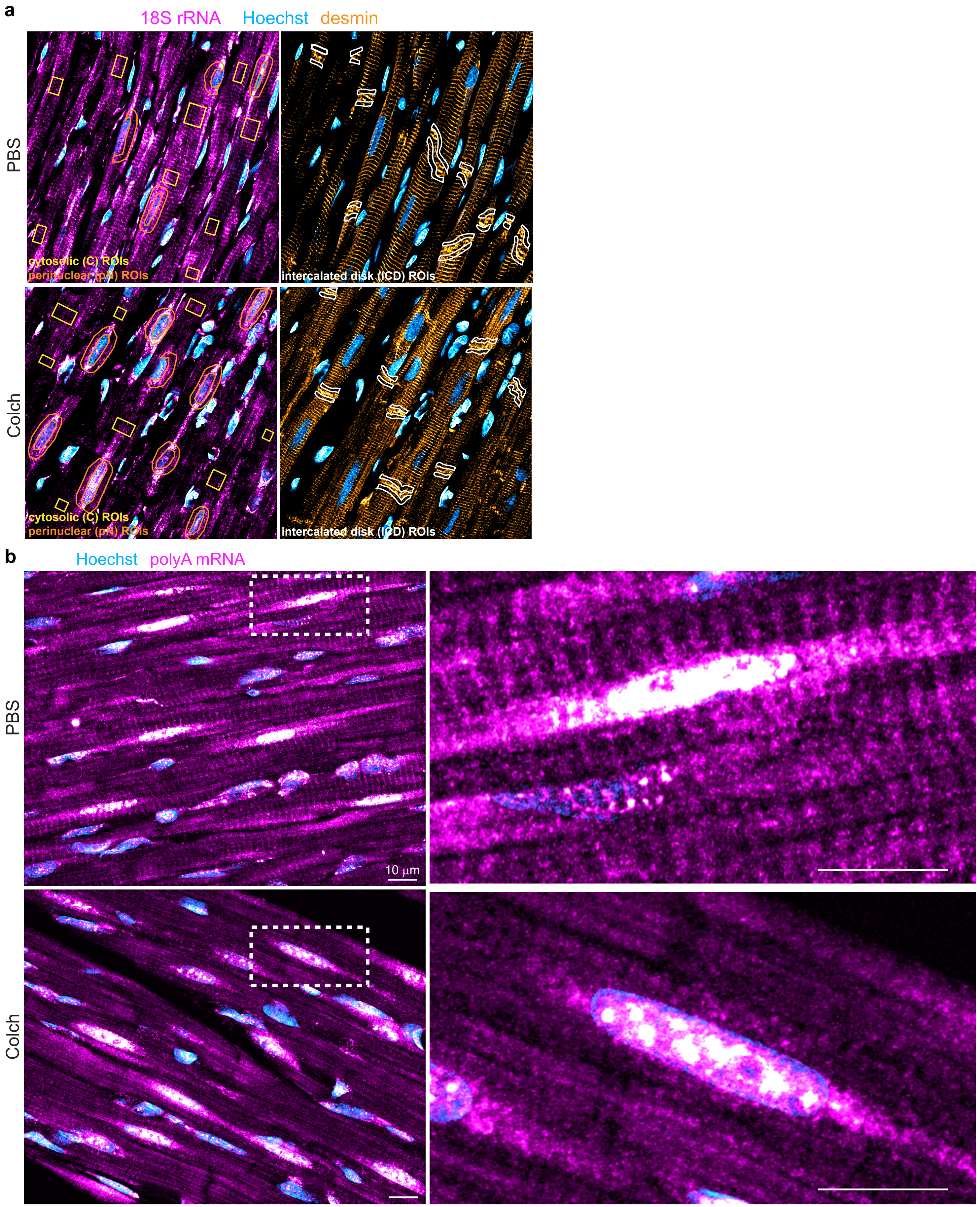

Supplementary Figure 6. Microtubule destabilization mislocalizes mRNA and ribosomes in vivo

a, Example ROI selection for analysis of rat heart tissue section stained for 18s rRNA and desmin (identical representative images from Fig. 4D and 4E, analysis shown in Fig. 4G). **b,** Representative images of polyA mRNA hybridized PBS-injected rat heart tissue section and Colch-injected rat heart tissue section. Region of zoomed-in images (right) indicated by white dotted box. Experiment was attempted more than 4 times, with variable conditions and results, as discussed in the Results section.

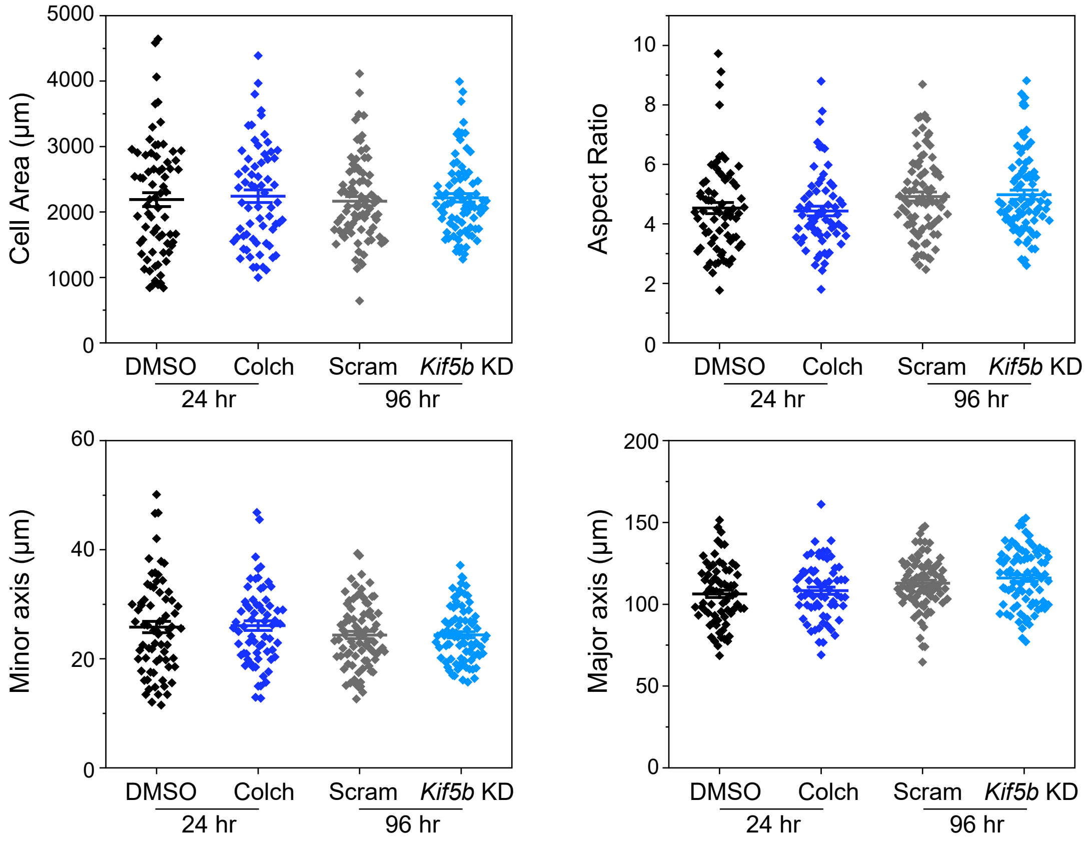

Supplementary Figure 7. ARVMs do not change dimensions after 96 hours in culture or upon Kif5b KD.
Cell morphology parameters after 24 hour DMSO (N = 4, n = 71) or colchicine (N = 4, n = 66) treatment, and in 96 hour shScramble (N = 4, n = 82) or shKif5B (N = 4, n = 83) treated ARVMs. For all graphs shown in figure, the mean line is shown, with whiskers denoting standard error (SE) from the mean. Source Data are provided as a Source Data file.

Supplementary Information

Supplementary Table 1: qPCR Primer sequences

Gene name	Primer 1 (5' > 3')	Primer 2 (5' > 3')
Myh6	GGACAATCCTAATGCAAACAAGG	TCCTGATGAACTTCCCAAAGC
Myh7	TGACCTGAAGCTGACCCA	CCTGCTCATCCTCAATCCTG
Tnni1	CATCTGCACAGGAACCAACA	GCTCTTCAGCATGAGTTTACG
Tnni3	GCCAACTACCGAGCCTATG	CTGCAGCATCAGAGTCTTCA
Nppa	GGTAGGATTGACAGGATTGGA	GCAGATTTGGCTGTTATCTTCG
Gapdh	AACCCATCACCATCTTCCAG	CCAGTAGACTCCACGACATA

Supplementary Table 2: Rat 18s rRNA custom smFISH probe set sequence

1	agagggaggtagacatcgac
2	cattgcgcgagagatggaag
3	gaaagagaagcgcgacaccg
4	acttagacatgcatggctta
5	cattcgcagtttcactgtac
6	gagcgaccaaaggaaccata
7	accacagttatccaagtagg
8	gggttggttttgatctgata
9	gatagggcagacgttcgaat
10	acggcgactaccatcgaaag
11	gttatttttcgtcactacct
12	attacagggcctcgaaagag
13	tccaatggatcctcgttaaa
14	tacgctattggagctggaat
15	acgagctttttaactgcagc
16	acactcagctaagagcatcg
17	tcaaagtaaacgcttcgggc
18	gcctgctttgaacactctaa
19	tcctattccattattcctag
20	cctcagttccgaaaaccaac
21	ttcttggcaaatgctttcgc
22	ccgactttcgttcttgatta

23	ggtatctgatcgtcttcgaa
24	ggaacccaaagactttggtt
25	tcagctttgcaaccatactc
26	cttccgtcaattcctttaag
27	tcccgtgttgagtcaaatta
28	atcaatctgtcaatcctgtc
29	cacccacggaatcgagaaag
30	aactaagaacggccatgcac
31	taaccagacaaatcgctcca
32	cagagtctcgttcgttatcg
33	gtcgcgtaactagttagcat
34	cttgtccctctaagaagttg
35	tgttattgctcaatctcggg
36	acatctaagggcatcacaga
37	aatggggttcaacgggttac
38	tggggaataattgcaatccc
39	cacttactgggaattcctcg
40	gtacaaagggcagggactta
41	cactaaaccatccaatcggt
42	agatagtcaagttcgaccgt
43	cacctacggaaaccttgtta
