# Supplementary material for: Microtubules orchestrate local translation to enable cardiac growth
Source: Nat Commun. 2021 Mar 11;12:1547. doi: 10.1038/s41467-021-21685-4 (PMC7952726; doi:10.1038/s41467-021-21685-4)
Supplement: Supplementary file 3 — Reporting Summary [file 41467_2021_21685_MOESM3_ESM.pdf]

nature research

Corresponding author(s): Benjamin Prosser

Last updated by author(s): 01/27/2021

Reporting Summary

Nature Research wishes to improve the reproducibility of the work that we publish. This form provides structure for consistency and transparency in reporting. For further information on Nature Research policies, see our Editorial Policies and the Editorial Policy Checklist.

Statistics

For all statistical analyses, confirm that the following items are present in the figure legend, table legend, main text, or Methods section.

n/a	Confirmed	
☐	☒	The exact sample size (n) for each experimental group/condition, given as a discrete number and unit of measurement
☐	☒	A statement on whether measurements were taken from distinct samples or whether the same sample was measured repeatedly
☐	☒	The statistical test(s) used AND whether they are one- or two-sided<br>*Only common tests should be described solely by name; describe more complex techniques in the Methods section.*
☐	☒	A description of all covariates tested
☐	☒	A description of any assumptions or corrections, such as tests of normality and adjustment for multiple comparisons
☐	☒	A full description of the statistical parameters including central tendency (e.g. means) or other basic estimates (e.g. regression coefficient) AND variation (e.g. standard deviation) or associated estimates of uncertainty (e.g. confidence intervals)
☐	☒	For null hypothesis testing, the test statistic (e.g. F, t, r) with confidence intervals, effect sizes, degrees of freedom and P value noted<br>*Give P values as exact values whenever suitable.*
☒	☐	For Bayesian analysis, information on the choice of priors and Markov chain Monte Carlo settings
☒	☐	For hierarchical and complex designs, identification of the appropriate level for tests and full reporting of outcomes
☒	☐	Estimates of effect sizes (e.g. Cohen's d, Pearson's r), indicating how they were calculated

Our web collection on statistics for biologists contains articles on many of the points above.

Software and code

Policy information about availability of computer code

Data collection	Image collection and Airyscan processing was performed using Zen Black software (Zeiss, Version 14.0.15.201). Image collection (NRVM smFISH data only) and deconvolution was performed using Zen Blue software (Zeiss, Version 2.5). LICOR Image studio (Version 5.2) was used to image western blots. QuantStudio 3 (Version v1.4.1) was used to collect qPCR amplification data.
Data analysis	ImageJ (Version 1.52n) was used for quantification of microscopy images. Image studio lite (Version 5.2) was used for western blot quantification. OriginPro (Version 2019, academic) was used for graphing and statistical analysis.

For manuscripts utilizing custom algorithms or software that are central to the research but not yet described in published literature, software must be made available to editors and reviewers. We strongly encourage code deposition in a community repository (e.g. GitHub). See the Nature Research guidelines for submitting code & software for further information.

Data

Policy information about availability of data

All manuscripts must include a data availability statement. This statement should provide the following information, where applicable:

- Accession codes, unique identifiers, or web links for publicly available datasets
- A list of figures that have associated raw data
- A description of any restrictions on data availability

Source data files are provided for all source data. Additional data that support the findings of this study are available from the corresponding author upon reasonable request.

Field-specific reporting

Please select the one below that is the best fit for your research. If you are not sure, read the appropriate sections before making your selection.

- [x] Life sciences
- [ ] Behavioural & social sciences
- [ ] Ecological, evolutionary & environmental sciences

For a reference copy of the document with all sections, see nature.com/documents/nr-reporting-summary-flat.pdf

Life sciences study design

All studies must disclose on these points even when the disclosure is negative.

Sample size	After a preliminary cohort was used to estimate variability, a power analysis was performed to estimate the number of additional mice necessary to complete the in vivo mouse studies. For imaging experiments, a sufficient number of cells were imaged to observe a clear distribution.
Data exclusions	One colchicine-injected mouse was excluded from the in vivo mouse hypertrophy study due to excessive weight loss.
Replication	All experiments where reproduced, typically in triplicate, and the number of biological replicates for each experiment are noted. Replication attempts were successful.
Randomization	Mice were randomly allocated based on the order that they were tagged for identification.
Blinding	Blinding was deemed unproductive as investigators could readily identify experimental groups based on stark phenotypes. As such, unbiased analytical approaches were used whenever possible to limit investigator bias.

Reporting for specific materials, systems and methods

We require information from authors about some types of materials, experimental systems and methods used in many studies. Here, indicate whether each material, system or method listed is relevant to your study. If you are not sure if a list item applies to your research, read the appropriate section before selecting a response.

Materials & experimental systems

n/a	Involved in the study	
	x	Antibodies
x		Eukaryotic cell lines
x		Palaeontology and archaeology
	x	Animals and other organisms
	x	Human research participants
x		Clinical data
x		Dual use research of concern

Methods

n/a	Involved in the study	
x		ChIP-seq
x		Flow cytometry
x		MRI-based neuroimaging

Antibodies

Antibodies used	Alpha-tubulin rabbit polyclonal (Millipore Sigma, SAB3501072-100UG);<br>Tubulin (DM1A) mouse monoclonal (Cell Signaling Technology, 3873S);<br>Desmin rabbit polyclonal (ThermoFisher, PA5-16705);<br>Kif5b rabbit monoclonal (Abcam, ab167429);<br>Rps6 (clone 54D2) mouse monoclonal (Cell Signaling Technology, 2317S);<br>Puromycin (clone 12D10) mouse monoclonal (Millipore Sigma, MABE343);<br>GAPDH rabbit polyclonal (Biolegend, Poly 6314);<br>Histone H3 rabbit polyclonal (Abcam, ab1791);<br>GAPDH mouse monoclonal (VWR, A01622-40);<br>Histone H3 mouse monoclonal (Abcam, ab24834);<br>Goat anti-rabbit IgG AF 647 (Life Technologies, A27040);<br>Goat anti-mouse IgG AF 488 (Life Technologies, A11001)
Validation	Alpha-tubulin rabbit polyclonal (Millipore Sigma, SAB3501072-100UG);<br>Tubulin (DM1A) mouse monoclonal (Cell Signaling Technology, 3873S);360 citations on Citeab, orthogonal validation https://www.cellsignal.co.uk/products/primary-antibodies/a-tubulin-dm1a-mouse-mab/3873?N=0+4294956287&Nrpp=200&No=5800&fromPage=plp<br>Desmin rabbit polyclonal (ThermoFisher, PA5-16705);Knockdown validated in Heffler et al. [PMID: 31822208]<br>Kif5b rabbit monoclonal (Abcam, ab167429);Knockdown validated in this manuscript

Rps6 (clone 54D2) mouse monoclonal (Cell Signaling Technology, 2317S);444 citations on Citeab https://www.citeab.com/antibodies/123215-2317-s6-ribosomal-protein-54d2-mouse-mab?des=4d8d40dfcb3076c5, Knockdown validated in Yano et al [PMID: 24557881]
Puromycin (clone 12D10) mouse monoclonal (Millipore Sigma, MABE343);Validated using no puromycin negative control
GAPDH rabbit polyclonal (Biolegend, Poly 6314);
Histone H3 rabbit polyclonal (Abcam, ab1791); Nuclear loading control and ChIP grade, 3328 citations on Citeab https://www.citeab.com/antibodies/763778-ab1791-anti-histone-h3-antibody-nuclear-loading-con?des=dbc569e5ef3eab3a
GAPDH mouse monoclonal (VWR, A01622-40);
Histone H3 mouse monoclonal (Abcam, ab24834);Nuclear loading control and ChIP grade, 35 citations on Citeab https://www.citeab.com/antibodies/763782-ab24834-anti-histone-h3-antibody-mabcam-24834-nuc?des=e1608f0371daaa69

Animals and other organisms

Policy information about studies involving animals; ARRIVE guidelines recommended for reporting animal research

Laboratory animals	8-12 week old, male Sprague Dawley rats were used for the isolation of primary adult ventricular myocytes;<br>1-2 day old litters (both male and female) of Sprague-Dawley rats were used to isolate neonatal ventricular myocytes;<br>8-12 week old male C57BL/6 wild type mice were used for in vivo colchicine and phenylephrine treatments
Wild animals	No wild animals were used in this study.
Field-collected samples	No field-collected samples were used in this study.
Ethics oversight	Animal care and use procedures were performed in accordance with the standards set forth by the University of Pennsylvania Institutional Animal Care and Use Committee and the Guide for the Care and Use of Laboratory Animals published by the US National Institutes of Health; protocols were approved by the University of Pennsylvania Institutional Animal Care and Use Committee.

Note that full information on the approval of the study protocol must also be provided in the manuscript.

Human research participants

Policy information about studies involving human research participants

Population characteristics	One human heart sample was used in this study: 64 year old Caucasian male with a BMI of 28.9, with history of one myocardial infarction in 1995 and hypertension.
Recruitment	Hearts are procured through cadaveric donation when organs are not suitable for transplantation. Non-failing donor hearts are characterized by having a left ventricle ejection fraction greater than 50%.
Ethics oversight	Procurement of human myocardial tissue was performed under protocols and ethical regulations approved by Institutional Review Boards at the University of Pennsylvania and the Gift-of-Life Donor Program (Pennsylvania, USA). Informed consent was obtained from subjects or relatives. Human studies were conducted in compliance with the principles of the Declaration of Helsinki.

Note that full information on the approval of the study protocol must also be provided in the manuscript.
